# Comparison of primary and secondary stroke outcomes between government and private healthcare institutions: insights from the SPRINT India trial

**DOI:** 10.3389/fstro.2026.1805422

**Published:** 2026-04-13

**Authors:** Shweta Jain Verma, Bhavya Nanda, Paramdeep Kaur, Arya Devi K. S., Deepti Arora, Aneesh Dhasan, P. N. Sylaja, Dheeraj Khurana, Vijaya Pamidimukkala, Biman Kanti Ray, Vivek Nambiar, Sanjith Aaron, Gaurav Mittal, Aparna R. Pai, Sankar Prasad Gorthi, Somasundaram Kumaravelu, Yerasu Muralidhar Reddy, Sunil Narayan, Nomal Chandra Borah, Rupjyoti Das, Girish Baburao Kulkarni, Vikram Huded, Thomas Mathew, Rohit Bhatia, Pawan Kumar Ojha, Jayanta Roy, Anand Girish Vaishnav, Arvind Sharma, Abhishek Pathak, Sanjeev Kumar Bhoi, Sudhir Sharma, Sulena Sulena, Aralikatte Onkarappa Saroja, Neetu Ramrakhiani, Madhusudhan Byadarahalli Kempegowda, Mahesh Kate, Tina George, Ivy Sebastian, Meenakshi Sharma, Rupinder Dhaliwal, Jeyaraj D. Pandian

**Affiliations:** 1 Department of Neurology, Christian Medical College, Ludhiana, Punjab, India; 2 Department of Obstetrics and Gynaecology, University of British Columbia, Vancouver, BC, Canada; 3 Department of Neurology, Sree Chithra Tirunal Institute for Medical Sciences and Technology, Thiruvananthapuram, Kerala, India; 4 Department of Neurology, Post Graduate Institute of Medical Education and Research, Chandigarh, India; 5 Department of Neurology, Lalitha Super Speciality Hospital, Guntur, Andhra Pradesh, India; 6 Department of Neurology, Bangur Institute of Neurosciences, Kolkata, West Bengal, India; 7 Department of Neurology, Amrita Institute of Medical Sciences, Kochi, Kerala, India; 8 Department of Neurology, Christian Medical College, Vellore, Tamil Nadu, India; 9 Department of Neurology, St. Stephens Hospital, New Delhi, India; 10 Department of Neurology, Kasturba Medical College and Hospital, Manipal, Karnataka, India; 11 Department of Neurology, Ramesh Hospital, Guntur, Andhra Pradesh, India; 12 Department of Neurology, CARE Hospital, Hyderabad, Telangana, India; 13 Department of Neurology, Jawaharlal Institute of Postgraduate Medical Education and Research, Puducherry, India; 14 Department of Neurology, Guwahati Neurological Research Centre, Dispur, Guwahati, India; 15 Department of Neurology, National Institute of Mental Health and Neurosciences, Bangalore, Karnataka, India; 16 Department of Neurology, Mazumdar Shaw Medical Centre, Narayana Hrudayalaya, Bangalore, Karnataka, India; 17 Department of Neurology, St. John's Medical College, Bangalore, Karnataka, India; 18 Department of Neurology, All India Institute of Medical Sciences, New Delhi, India; 19 Department of Neurology, Grant Government Medical College and Sir J.J. Group of Hospitals College, Mumbai, India; 20 Department of Neurology, Institute of Neurosciences, Kolkata, West Bengal, India; 21 Department of Neurology, Vadodara Institute of Neurosciences, Vadodara, Gujarat, India; 22 Department of Neurology, Zydus Hospital, Ahmedabad, Gujarat, India; 23 Department of Neurology, Institute of Medical Sciences, Banaras Hindu University, Varanasi, Uttar Pradesh, India; 24 Department of Neurology, All India Institute of Medical Sciences, Bhubaneswar, Odisha, India; 25 Department of Neurology, Indira Gandhi Medical College and Hospital, Shimla, Himachal Pradesh, India; 26 Department of Neurology, Guru Gobind Singh Medical College, Faridkot, Punjab, India; 27 Department of Neurology, KLE Academy of Higher Education and Research, Belagavi, Karnataka, India; 28 Department of Neurology, Fortis Escorts Hospital, Jaipur, Rajasthan, India; 29 Department of Neurology, BGS Global Hospital, Bangalore, Karnataka, India; 30 Department of Medicine, University of Alberta, Edmonton, AB, Canada; 31 Non-Communicable Diseases Section, Indian Council of Medical Research, New Delhi, India

**Keywords:** functional outcomes, government and private hospitals, medication adherence, mHealth intervention, SPRINT INDIA trial, stroke secondary prevention

## Abstract

**Introduction:**

Stroke recurrence and vascular events remain major contributors to post-stroke mortality in India, where care is delivered through heterogeneous government and private healthcare systems. This *post-hoc* analysis of the Secondary Prevention with a Structured Semi-Interactive Stroke Prevention Package in India (SPRINT INDIA) trial compared primary and secondary stroke outcomes between government and private hospitals.

**Methods:**

SPRINT INDIA was a multicentre randomized controlled trial conducted across 31 centers (18 private, 13 government) within the Indian Stroke Clinical Trial Network (INSTRuCT). Adults with sub-acute stroke were randomized to a structured mHealth-supported secondary prevention intervention or standard care and followed for 1 year. The primary outcome was a composite of recurrent stroke, high-risk transient ischemic attack, acute coronary syndrome, and all-cause mortality. Secondary outcomes included functional status (modified Rankin Scale), behavioral risk factors, medication adherence, body mass index, physical activity, and laboratory measures of vascular risk. Outcomes were compared between government and private hospitals using adjusted regression models.

**Results:**

Among 4,298 randomized patients, 3,038 completed 1-year follow-up (59.8% private; 40.2% government). There was no significant difference was observed in the composite primary outcome between private and government institutions (2.8 vs. 3.7%; *p* = 0.215). Private hospitals demonstrated non-significantly lower adjusted odds of the primary outcome and higher odds of good functional recovery. The intervention was associated with improved functional outcomes and reductions in systolic blood pressure and fasting blood glucose in government hospitals, while private hospitals showed greater improvements in medication adherence, smoking and alcohol cessation, and body mass index.

**Conclusions:**

Stroke outcomes at 1 year were broadly comparable across healthcare sectors; however, the intervention demonstrated context-specific benefits, improving risk factors and functional recovery in government hospitals and reinforcing behavioral adherence in private hospitals. These findings highlight the importance of tailoring secondary stroke prevention strategies to healthcare system context.

**Clinical trial registration:**

http://ctri.nic.in, identifier: CTRI/2017/09/009600.

## Introduction

Stroke is a major global health challenge and is recognized as a major cause of death and disability across the world (World Health Organization, 2025). Recurrent stroke along with coronary artery disease (CAD) is the major cause of death which accounts to 60% of mortality after 1-month post stroke (Pandian et al., 2023). Public awareness about stroke symptoms, and management of risk factors and importance of early intervention remains low. Reports from various studies show rapidly increasing incidence rates over the past few decades in India with rates of stroke recurrence at 1 year varying from 7 to 20% (Verma et al., 2022).

In India, the healthcare sector is predominantly divided into government and private institutions and the dichotomy has been a subject of extensive debate, concerning their efficacy and in delivering quality health care and improving patient outcomes (Owolabi et al., 2021; Rangamani et al., 2023; Verma et al., 2022). According to National Stroke Registry Programme, data collected from five geographical areas from different regions showed hypertension (HTN) is the most common risk factor among stroke patients in India (Rangamani et al., 2023). Among the population only 50% of the cases with known HTN were on treatment and less than half of them had their blood pressure under control (Bhate-Deosthali et al., 2011). Noncompliance to medications along with other risk factors like alcohol consumption, smoking, increased Body Mass Index (BMI), and Fasting Blood Sugar (FBS) are associated with recurrent stroke in patients (Pandian et al., 2023).

The Indian Stroke Clinical Trial Network (INSTRuCT) was established in 2017 with funding from the Indian Council of Medical Research (ICMR), the apex body in India responsible for the formulation, coordination, and promotion of biomedical research. The primary objective of INSTRuCT was to develop a state-of-the-art national stroke clinical trial network capable of conducting high-quality pharmacological and non-pharmacological stroke trials relevant both nationally and globally (Pandian et al., 2021).

The Secondary Prevention with a Structured Semi-Interactive Stroke Prevention Package in India (SPRINT INDIA) trial was the first major study conducted under the INSTRuCT network. It was a multicentre, randomized controlled trial implemented across 31 centers (including both government and private institutions) in India. Adult patients with a first-ever stroke and access to a mobile phone were randomly allocated in a 1:1 ratio to intervention or control groups using a centralized, in-house, web-based randomization system managed by research coordinators at each center.

The trial aimed to evaluate the effect of a structured semi-interactive stroke prevention package in patients with sub-acute stroke to reduce recurrent stroke, myocardial infarction, and death. The first patient was randomized in April 2018, and between April 28, 2018, and November 30, 2021, a total of 5,640 patients were screened for eligibility. A total of 4,298 patients were randomized to either the intervention group (*n* = 2,148) or control group (*n* = 2,150; Pandian et al., 2023).

Intervention group received regular short SMS messages, videos promoting risk factor control and medication adherence, and an educational workbook in one of 12 languages, while the control group received standard care (Pandian et al., 2023). The primary outcome was a composite of recurrent stroke, high-risk transient ischemic attack (TIA), acute coronary syndrome (ACS), and death at 1 year after randomization. Secondary outcomes included modified Rankin Scale (mRS), change in BMI categorized into underweight, normal, overweight and obese, physical activity total metabolic equivalent (min per week), current smoking, current alcohol intake, and medication non-compliance and reasons for medication non-compliance (Kate et al., 2020a). The laboratory-measured secondary outcomes were systolic and diastolic blood pressure (SBP and DBP; mm Hg), FBS (mg/dL), low-density lipoprotein (LDL) Total cholesterol (TC; mg/dL), and Triglycerides (TGs; mg/dL) at 1 year after randomization (Pandian et al., 2023).

This paper aims to compare the primary and secondary outcomes between government and private hospitals in relation to baseline characteristics at 1 year in the SPRINT INDIA trial. Understanding the differences in outcomes between these two healthcare settings can provide valuable insights into the effectiveness of the intervention package and guide future implementation strategies. This *post hoc* study was done with the following objectives.

## Objectives

1. Primary objective:

a. To compare and evaluate the primary health outcomes (composite endpoint of recurrent stroke, high-risk transient ischemic attack acute coronary syndrome, and all-cause mortality at 1 year after randomization) between government and private healthcare institutions over 1 year in the SPRINT India trial.

2. Secondary objectives:

a. To compare and evaluate secondary health outcomes (change in BMI, physical activity total metabolic equivalent (min per week; Pandian et al., 2023), current smoking, current alcohol intake, mRS, and medication non-compliance (Pandian et al., 2023). The laboratory-measured secondary outcomes were SBP and DBP (mm Hg), FBS (mg/dL), LDL, TC (mg/dL), and TGs (mg/dL) at 1 year after randomization) between government and private institutions over 1 year.
b. To analyze the baseline characteristics (e.g., demographic characteristics, stroke subtype, risk factor profile, SBP and DBP, FBS, lipid profile, total physical activity metabolic equivalent, and medication non-compliance), as well as imaging data (including vascular evaluation of intracranial and extra cranial arteries and the location of infarct or hemorrhage), of participants in government vs. private institutions and to assess their influence on primary and secondary outcomes.

3. Exploratory objective:

a. To explore potential reasons for any observed differences in outcomes between government and private institutions, including but not limited to healthcare infrastructure, provider expertise, and patient follow-up practices.

## Methods

**Figure 1:**
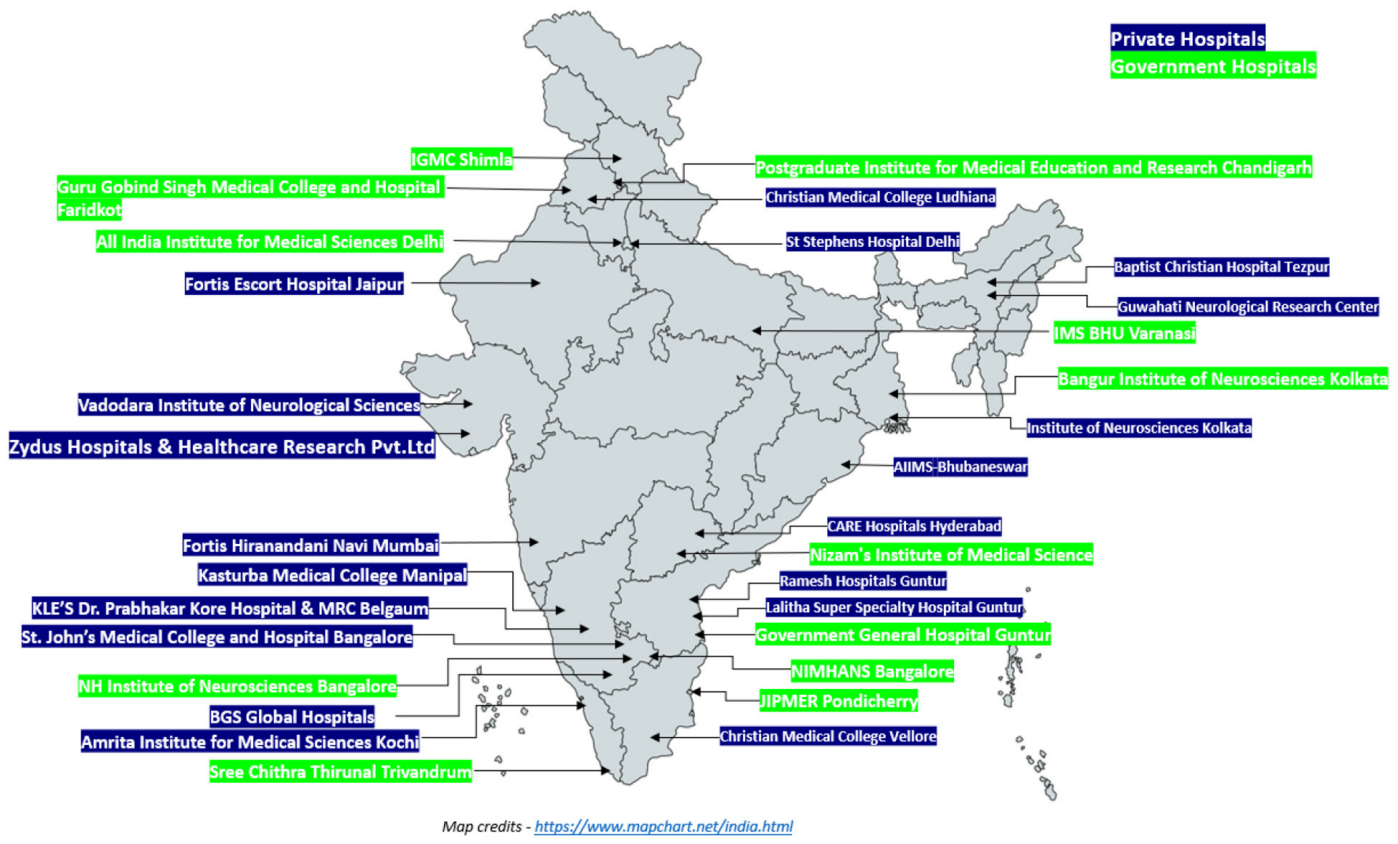
Participating government and private hospitals across India in the SPRINT India trial.

SPRINT INDIA, a multicentre RCT conducted among patients with sub-acute stroke, was implemented under the INSTRuCT. This network consists of 31 stroke centers distributed across India, representing a diverse mix of healthcare settings, including 18 private hospitals, and 13 government hospitals presented in Figure 1 (Pandian et al., 2023). The comparison between government and private institutions was not part of the original randomization and therefore represents a *post-hoc* exploratory analysis.

Eligible participants were adults aged 18 years and above who had experienced an index stroke and presented between 2 days and 3 months after symptom onset. Patients were enrolled only if they were able to read and engage with the stroke educational workbook and possessed a functional mobile phone capable of receiving SMS messages and videos (Rangamani et al., 2023). Using a secure, computer-generated web-based randomization process, participants were assigned in a 1:1 ratio to either the intervention group or the control group (Pandian et al., 2023).

### Intervention

The intervention consisted of a comprehensive package that included 68 structured SMS messages, six video messages each lasting approximately 2–4 min, and patient-friendly workbooks (Pandian et al., 2023). These materials were delivered over a 1-year period to participants in the intervention arm and were made available in 12 widely spoken Indian languages to ensure accessibility and cultural relevance. The SMS and videos were carefully designed to highlight potential problems related to stroke risk factors while simultaneously conveying messages of encouragement and hope, with the overarching aim of promoting sustainable behavioral changes and preventing stroke recurrence.

At the baseline visit, each participant received a workbook that featured a variety of interactive stroke-prevention activities, including board games, matching exercises, true-or-false questions with answers, and simple physical activity prompts (Pandian et al., 2023). To facilitate monitoring and engagement, every SMS and video message included a dedicated phone number that patients or their caregivers were asked to call to confirm receipt; each call served as a documented acknowledgment of the delivered message (Pandian et al., 2023).

As part of the structured follow-up, patients in the intervention group underwent vascular assessments at both 6 months and 1 year. Participants in the control group also completed these scheduled follow-up visits in addition to receiving standard routine care. Further detailed information regarding the design, development, and creation of the intervention components is available in the research article describing the intervention development process (Kate et al., 2020a). Confirmation of receipt of the SMS messages and videos among patients in the intervention group was low, with only 17% acknowledging receipt.

### Baseline characteristics and evaluations

The baseline assessment consisted of a comprehensive set of variables that included demographic details, clinical stroke subtype, an extensive profile of vascular and lifestyle-related risk factors, BP measurements, FBS, lipid parameters, total physical activity expressed in metabolic equivalents, as well as information on medication non-compliance. Definitions for behavioral factors were standardized: a “current smoker” was identified as any individual who had used smoked tobacco within the 12 months preceding enrolment in the trial, while “current alcohol intake” referred to alcohol consumption during the same period (Pandian et al., 2023).

Medication non-compliance was evaluated using a structured self-report questionnaire that explored the underlying reasons for missed medication doses, thereby enabling a nuanced understanding of adherence barriers. Educational attainment, captured through the trial's Case Report Form (CRF), was categorized in detail as follows: less than primary school education (< 5th standard); completion of primary school (up to 5th standard); completion of middle school (up to 8th standard); completion of secondary school (up to 10th standard); completion of high school (up to 12th standard); attainment of a diploma, college, or university degree; possession of a postgraduate qualification; and an “unknown” category for cases where information could not be ascertained (Pandian et al., 2023).

### Outcomes and follow-up assessments

The primary outcome comprised a composite measure that included recurrent stroke, high-risk TIA, ACS, and all-cause mortality. A range of secondary outcomes was also evaluated, capturing broader aspects of recovery and health behavior. These included changes in BMI, total physical activity expressed as metabolic equivalent minutes per week, current smoking status, current alcohol consumption, functional status measured by the mRS, and levels of medication non-compliance at the 1-year mark. Outcome assessments for both the intervention and control groups were conducted 1 year after randomization to ensure uniform follow-up. Functional outcomes (mRS) were assessed at 1 year using standardized definitions as per the trial protocol. Outcome assessors were the treating physicians (center principal investigators), who were masked to group allocation across all participating centers.

An interim futility analysis, undertaken by the Data and Safety Monitoring Board (DSMB), ultimately led to the early termination of the trial (Pandian et al., 2023). The trial was halted primarily due to funding limitations and lack of demonstrable effectiveness (Pandian et al., 2023). The DSMB's recommendation was based on a lower-than-expected conditional power and a considerably smaller number of primary vascular outcome events than originally anticipated, which together reduced the likelihood that extending the trial would yield meaningful or conclusive results.

### Statistical analysis

A chi-square test was used to find out the difference between patients in the private and government hospital based on group allocation, baseline characteristics, mRS, and baseline behavioral history such as alcohol intake, smoking, chewed tobacco, drug addiction, and medication compliance. An independent sample *t*-test was used to find out the difference between private and government hospitals based on SBP, DBP, FBS, BMI, LDL, high density lipids HDL, TCs, TGs, and physical activity score.

The comparison between government and private hospitals was conducted as a *post-hoc* exploratory analysis. The original SPRINT INDIA trial was powered to assess differences between intervention and control groups, not to detect differences between hospital sectors; therefore, these analyses were not based on a pre-specified sample size calculation for sector comparison (Pandian et al., 2021).

The primary outcomes at 1 year in the SPRINT-INDIA trial, which includes a composite end point of recurrent stroke, high-risk TIA, ACS, and all-cause mortality, were assessed using chi-square tests. The secondary outcomes assessed at 1 year after randomization were changes in BMI, physical activity total metabolic equivalent (min per week), current smoking, current alcohol intake, mRS, and medication non-compliance. The laboratory-measured secondary outcomes were SBP and DBP (mm Hg), FBS (mg/dL), LDL (mg/dL), and triglycerides (mg/dL) at 1 year after randomization.

At 1 year follow-up, the comparison between the two types of hospitals for the primary and secondary outcomes in the SPRINT-INDIA trial was analyzed using the chi-square test for the categorical variables and the *t*-test for the continuous variables, both overall at 1 year and within the intervention and control groups. The comparison between the two types of hospitals in terms of the patients' work status prior and after stroke analyzed using the chi-square test. Generalized Estimation Equation (GEE) analysis included an interaction term (type of institutions^*^group) to assess outcomes between groups, adjusting for age, education, stroke characteristics, medical history, and NIH Stroke Scale, while accounting for within-center correlation using an unstructured working correlation structure. For the categorical variables adjusted odds ratio and 95% confidence interval were presented. For continuous variables, β coefficient with 95% confidence interval was presented. All the analyses were conducted using a complete-case approach, including only participants with available data at the respective follow-up time points. No imputation method were applied for missing data. All *p* values < 0.05 was considered significant. The statistical analysis was conducted using SPSS version 29.0.

## Results

**Figure 2:**
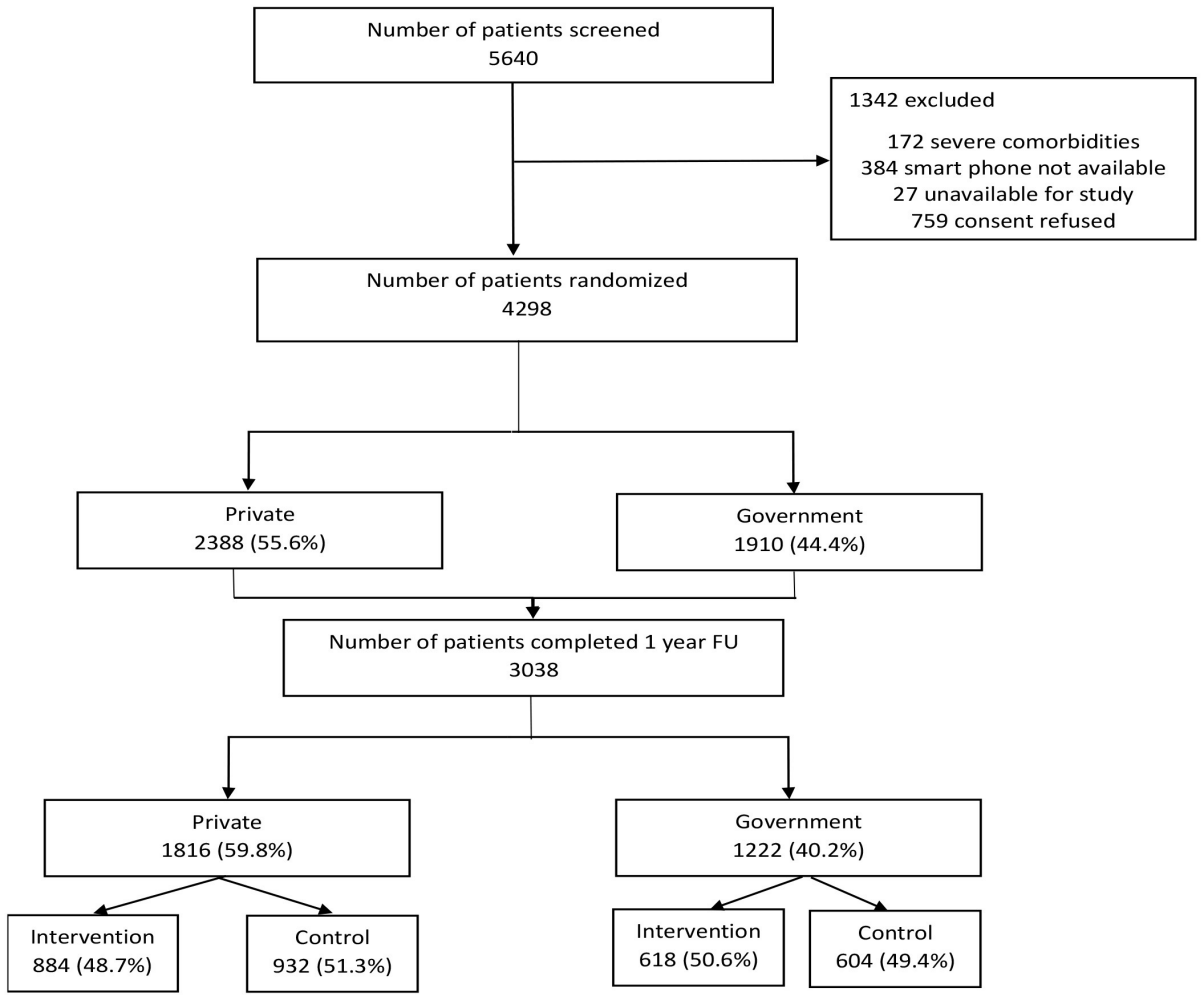
Consort diagram based on the SPRINT INDIA trial participants and their Hospital.

Among 5,640 patients screened, 4,298 patients were randomized out of which 2,388 (55.6%) were in private hospitals and 1,910 (44.4%) were in government hospitals presented in Figure 2. Out of these only 3,038 patients completed 1 year follow up among which 59.8% were private and 40.2% were in government hospitals. A total of 620 participants did not complete the 6-month follow-up, and an additional 595 participants did not complete the 1-year follow-up due to early termination of the trial for futility following the interim analysis. Furthermore, 45 participants were lost to follow-up prior to the 1-year assessment.

### Difference between type of hospitals based on baseline characteristics

Table 1 shows the data for baseline characteristics at the time of start of study. More than half of the patients in both government and private hospitals had less than high school education [717 (58.7%) and 926 (50%) respectively; *p* < 0.001]. Ischemic strokes were more prevalent in private hospitals than government hospitals [1,579 (86.9%) vs. 974 (79.7%); *p* < 0.001].

**Table 1:** Difference between type of hospital in which the patients admitted and their baseline characteristics.

Baseline characteristic variables	Type of hospital		*p* value
	Private (*n* = 1,816)	Government (*n* = 1,222)	
Group allocation			
Intervention group	884 (48.7%)	618 (50.6%)	0.306
Control group	932 (51.3%)	604 (49.4%)	
Gender			
Male	1,305 (71.9%)	900 (73.6%)	0.279
Female	511 (28.1%)	322 (26.4%)	
North/South			
North	723 (39.8%)	549 (44.9%)	0.005
South	1,093 (60.2%)	673 (55.1%)	
Age			
≤ 50 years	614 (33.8%)	442 (36.2%)	0.181
>50 years	1,202 (66.2%)	780 (63.8%)	
Region			
Urban	988 (54.4%)	632 (51.7%)	0.146
Rural	828 (45.6%)	590 (48.3%)	
Place of residence			
Own home—independent	1,619 (89.2%)	1,017 (83.2%)	< 0.001
Own home—with financial assistance/Loan	19 (1%)	15 (1.2%)	
Family member's home	7 (0.4%)	9 (0.7%)	
Another hospital	1 (0.1%)	0 (0%)	
High care facility (e.g., nursing home, residential home)	0 (0%)	2 (0.2%)	
Intermediate care facility (e.g. hostel, shelter, and community based care)	170 (9.4%)	179 (14.6%)	
Education			
No schooling	95 (5.3%)	96 (7.9%)	< 0.001
< High school	926 (50%)	717 (58.7%)	
≥High school	785 (43.2%)	404 (33.1%)	
Type of stroke			
Ischemic	1,579 (86.9%)	974 (79.7%)	< 0.001
Hemorrhagic	237 (13.1%)	248 (20.3%)	
TOAST classification			
Large artery atherosclerosis	462 (30.4%)	340 (35.9%)	0.007
Cardioembolism	175 (11.5%)	116 (12.2%)	
Small artery occlusion	476 (31.3%)	243 (25.7%)	
Other	408 (26.8%)	248 (26.2%)	
OCSP classification (Oxford community stroke classification)			
Total anterior circulation syndrome	134 (8.5%)	221 (22.7%)	< 0.001
Partial anterior circulation syndrome	863 (54.7%)	413 (42.4%)	
Posterior circulation syndrome	378 (24%)	204 (21%)	
Lacunar syndrome	203 (12.9%)	135 (13.9%)	
Medical history			
Hypertension	1,279 (70.4%)	786 (64.3%)	< 0.001
Diabetes mellitus	789 (43.4%)	466 (38.1%)	0.004
Previous TIA	36 (2%)	43 (3.5%)	0.032
Coronary artery disease (includes myocardial infarction and angina)	199 (11%)	111 (9.1%)	0.013
Dyslipidaemia	359 (19.8%)	223 (18.2%)	0.450
Non valvular atrial fibrillation	29 (1.6%)	35 (2.9%)	0.005
Valvular heart disease	40 (2.2%)	27 (2.2%)	0.019
Symptomatic intracranial atherosclerosis	45 (2.5%)	27 (2.2%)	0.263
Symptomatic extra cranial atherosclerosis	20 (1.1%)	20 (1.6%)	0.128
Epilepsy/seizure disorder	41 (2.3%)	22 (1.8%)	0.589
Obesity	168 (9.3%)	52 (4.3%)	< 0.001
Other major health condition(s) that limits daily activity	87 (4.8%)	65 (5.3%)	0.784
Medication history			
Antiplatelet	1,380 (76%)	907 (74.3%)	0.397
Anticoagulants	221 (12.2%)	108 (8.9%)	0.005
Cholesterol reducing medications	1,376 (75.9%)	995 (81.4%)	0.001
Diabetic medications	704 (38.8%)	392 (32.7%)	0.001
Antihypertensive	1,184 (65.2%)	845 (69.2%)	0.040

According to TOAST classification large artery atherosclerosis prevalence is more in government hospitals 340 (35.9%) than private institutions 462 (30.4%) but according to OCSP classification partial anterior circulatory syndrome is reported to be more in private than government institutions [863 (54.7%) vs. 413 (42.4%); *p* < 0.001 respectively]. Majority of the patients admitted in private hospitals had HTN [1,279 (70.4%)], diabetes mellitus [789 (43.4%)], dyslipidemia [359 (19.8%)], coronary artery disease [199 (11.0%)], and obesity [168 (9.3%)] as compared to government hospitals. On the other hand, most of the patients in government hospitals as compared to private hospitals had TIA (3.5 vs. 2%; *p* = 0.032) and non-valvular atrial fibrillation (2.9 vs. 1.6%; *p* = 0.005).

The proportion of patients in private hospitals as compared to government hospitals were taking more medications like diabetic medications [704 (38.8%) vs. 392 (32.7%); *p* = 0.001] and anticoagulants [221 (12.2%) vs. 108 (8.9%); *p* = 0.005]. On the other hand, majority of the patients in government hospitals as compared to private hospitals had taken antihypertensive medications [845 (69.2%) vs. 1,184 (65.2%); *p* = 0.040].

Even though there exists no significant difference in terms of gender (*p* = 0.279) and age (*p* = 0.181) major portions of patients in private and government hospitals were males [1,305 (71.9%) vs. 900 (73.6%)] with more than 50 years old [1,202 (66.2%) vs. 780 (63.8%)].

### Differences based on baseline and behavioral history

Table 2 shows the baseline and behavioral history in patients among the private and government hospitals. The majority of the private hospital patients admitted had a good mRS [1,106 (60.9%)]. Most of the government hospital patients were using alcohol [265 (21.7%) vs. 333 (18.3%); *p* = 0.047] and tobacco smoking [269 (22%) vs. 249 (13.7%); *p* < 0.001] compared to private school patients. BMI, LDL, total cholesterol, TGs, and fasting blood sugar were high in private hospital patients (25.574 ± 0.14, 105.513 ± 9.22, 173.844 ± 4.66, 154.529 ± 2.81, and 138.1406 ± 0.53, respectively). On the other hand, SBP, DBP, and HDL were high in government hospital patients (134.961 ± 8.58, 83.411 ± 0.41, and 50.352 ± 4.51, respectively).

**Table 2:** Comparison of the type of hospital in which the patients' admitted with their baseline behavioral history.

Baseline behavioural history variables	Type of hospital		*p*-value
	Private (*n* = 1,816)	Government (*n* = 1,222)	
Modified Rankin scale			
0–2	1,106 (60.9%)	719 (58.8%)	0.254
3–5	710 (39.1%)	503 (41.2%)	
Alcohol intake			
Never	1,347 (74.2%)	880 (72%)	0.047
Current user	333 (18.3%)	265 (21.7%)	
Past history	136 (7.5%)	77 (6.3%)	
Smoked tobacco			
Never	1,390 (76.5%)	842 (68.9%)	< 0.001
Current user	249 (13.7%)	269 (22.0%)	
Past history	177 (9.7%)	111 (9.1%)	
Chewed tobacco			
Never	1,635 (90%)	1,111 (90.9)	0.436
Current user	143 (7.9%)	82 (6.7%)	
Past history	38 (2.1%)	29 (2.4%)	
Medication non-compliance			
No	2,036 (85.3%)	1,635 (85.6%)	0.752
Yes	352 (14.7%)	275 (14.4%)	
Drug addiction			
Never	1,809 (99.6%)	1,218 (99.7%)	0.123
Current user	7 (0.4%)	2 (0.2%)	
Past history	0 (0%)	2 (0.2%)	
Blood pressure and fasting blood glucose			
Systolic blood pressure, mm Hg	133.06 ± 17.41	134.96 ± 18.58	0.004
Diastolic blood pressure, mm Hg	82.48 ± 10.45	83.41 ± 10.41	0.016
Fasting blood glucose, mg/dL	138.14 ± 60.53	129.31 ± 54.12	< 0.001
Lipid profile, mg/dL			
LDL	105.51 ± 39.22	92.14 ± 42.89	< 0.001
HDL	41.95 ± 14.38	50.35 ± 24.51	< 0.001
Total cholesterol	173.84 ± 44.66	167.81 ± 48.79	< 0.001
Triglycerides	154.52 ± 92.81	132.51 ± 75.76	< 0.001
BMI and physical activity score			
BMI, kg/m^2^	25.57 ± 4.14	24.12 ± 3.50	< 0.001
Physical activity score, min	5,831.20 ± 4,972.08	6,201.65 ± 5,168.29	0.025

### Difference between the hospitals on the basis of primary outcome at 1 year of SPRINT India trial

At 1 year the study focused on the 3,038 patients that were followed up. No significant differences observed in the primary outcomes between the private and government hospitals as seen by the composite primary outcome [private: 50 (2.8%) vs. government: 45 (3.7%); *p* value 0.215; Table 3].

**Table 3:** At 1 year comparison between type of hospital in which the patients admitted with primary outcomes in SPRINT INDIA Trial (*n* = 3,038).

Primary outcomes at 1 year	Type of hospital		*p*-value
	Private (*n* = 1,816)	Government (*n* = 1,222)	
Composite primary outcome	50 (2.8%)	45 (3.7%)	0.215
High-risk TIA	4 (0.2%)	8 (0.7%)	0.061
Ischemic stroke	32 (1.8%)	31 (2.5%)	0.142
Intracerebral hemorrhage	3 (0.2%)	0 (0%)	0.155
Acute coronary syndrome	8 (0.4%)	5 (0.4%)	0.897
Death	3 (0.2%)	1 (0.1%)	0.534

### Difference between the hospitals on the basis of secondary outcome at 1 year

Table 4 presents the comparison of secondary outcomes based on type of hospital. Majority of the private hospital patients had a good mRS score 1,720 (94.7%). The higher proportion of patients in private hospitals consumed alcohol [private: 103 (5.7%) vs. government: 35 (2.9%); *p* < 0.001], smoked [private: 89 (4.9%) vs. government: 35 (2.9%); *p* < 0.001] than in government hospitals which is in contrast to the baseline characters where higher number of patients in government hospitals consumed alcohol and smoked tobacco. Greater number of patients in private hospitals chewed tobacco than government hospitals [private: 68 (3.7%) vs. government hospital: 16 (1.3%); *p* < 0.001]. Fasting blood glucose, LDL levels, TGs levels, and BMI were found to be higher in private hospital patients (119.324 ± 0.35, 87.113 ± 3.12, 141.67 ± 1.5, 25.604 ± 0.50 respectively) whereas HDL levels were higher in government hospital patients (51.81 + 22.68) which is similar to the baseline characters in the comparison groups (Table 4).

**Table 4:** At 1 year comparison between type of hospital in which the patients admitted with secondary outcomes in SPRINT INDIA Trial (*n* = 3,038).

Secondary outcomes at 1 year	Type of hospital		*p*-value
	Private (*n* = 1,816)	Government (*n* = 1,222)	
mRS outcome			
Good outcome (0–2)	1,720 (94.7%)	1,086 (88.9%)	< 0.0001
Bad outcome (3–5)	96 (5.3%)	136 (11.1%)	
Alcohol intake			
Never	1,407 (77.5%)	874 (71.5%)	< 0.0001
Current	107 (5.9%)	35 (2.9%)	
Past history	302 (16.6%)	313 (25.6%)	
Smoking intake			
Never	1,498 (82.5%)	901 (73.7%)	< 0.0001
Current	92 (5.1%)	37 (3%)	
Past history	226 (12.4%)	284 (23.2%)	
Chewing tobacco			
Never	1,681 (92.6%)	1,142 (93.5%)	< 0.0001
Current	68 (3.7%)	16 (1.3%)	
Past history	66 (3.6%)	64 (5.2%)	
Drug addiction			
Never	1,811 (99.8%)	1,216 (99.5%)	0.208
Current	0 (0%)	1 (0.1%)	
Past history	3 (0.2%)	5 (0.4%)	
Medication non-compliance			
No	1,655 (91.1%)	1,130 (92.5%)	0.191
Yes	161 (8.9%)	92 (7.5%)	
Blood pressure and fasting blood glucose			
Systolic blood pressure, mm Hg	130.74 ± 14.7	130.78 ± 13.13	0.941
Diastolic blood pressure, mm Hg	82.32 ± 9.07	82.85 ± 7.63	
Fasting blood glucose, mg/dL	119.32 ± 40.35	114.96 ± 36.55	0.004
Lipid profile, mg/dL			
LDL	87.11 ± 33.12	81.88 ± 31.67	< 0.0001
HDL	42.88 ± 13.2	51.81 ± 22.68	< 0.0001
Total cholesterol	152.96 ± 38.28	153.88 ± 40.76	0.567
TGs	141.6 ± 71.5	126.95 ± 61.96	< 0.0001
BMI and physical activity score			
BMI, kg/m^2^	25.60 ± 4.50	24.06 ± 3.67	< 0.0001
Physical activity score, min	4,852.35 ± 4,038.08	4,587.53 ± 3,648.34	0.066

## Subgroup analysis

### Comparison of intervention and control groups—stratified by type of hospitals

At 1 year, there were no significant difference in primary outcome between intervention and control group in patients who were admitted to private as well as government hospitals. There was no patient who had high risk of TIA in intervention group as compared to control group in private hospitals [intervention: 0 (0%); control: 4 (0.4%); *p* = 0.051; Table 5].

**Table 5:** At 1 year difference between the intervention and control group stratified by type of hospitals in which the patients were admitted (*n* = 3,038).

SPRINT India trial primary outcomes	Private hospital (*n* = 1,816)		*P* value	Government hospital (*n* = 1,222)		*P* value
	Intervention (*n* = 884)	Control (*n* = 932)		Intervention (*n* = 618)	Control (*n* = 604)	
Composite primary outcome	23 (2.6%)	27 (2.9%)	0.70	25 (4.1%)	20 (2.1%)	0.496
High risk TIA	0 (0.0%)	4 (0.4%)	0.06	5 (0.8%)	3 (0.5%)	0.224
Ischemic Stroke	13 (1.5%)	19 (2.0%)	0.358	16 (2.6%)	15 (2.5%)	0.907
Intracerebral hemorrhage	3 (0.3%)	0 (0%)	0.115	0 (0%)	0 (0%)	
Acute coronary syndrome	5 (0.6%)	3 (0.3%)	0.207	3 (0.5%)	2 (0.3%)	0.316
Death	2 (0.2%)	1 (0.1%)	0.365	1 (0.2%)	0 (0%)	0.506

Table 6 presents comparison of intervention and control group when stratified by private and government hospitals. The greater number of patients in control group has poor functional outcome in control group as compared to intervention group in government hospitals [intervention: 57 (9.2%); control: 79 (13.1%); *p* = 0.032]. A smaller proportion of patients in private hospitals reported alcohol use in the intervention group compared with the control group (3.7 vs. 7.5%; *p* = 0.002). Similarly, current smoking was less frequent among intervention recipients (3.6 vs. 6.1%; *p* = 0.027), and medication non-compliance was significantly lower in the intervention group (6.1 vs. 11.5%; *p* < 0.001). There were no significant differences between the intervention and control groups in blood pressure, fasting blood glucose levels, lipid profiles, BMI and physical activity scores among patients admitted to both private and government hospitals.

**Table 6:** At 1 year difference between the intervention and control group stratified by type of hospitals in which the patients were admitted (*n* = 3,038).

Categories	Private hospital (*n* = 1,816)		*P* value	Government hospital (*n* = 1,222)		*P* value
	Intervention (*n* = 884)	Control (*n* = 932)		Intervention (*n* = 618)	Control (*n* = 604)	
mRS outcome						
Good outcome (0–2)	833 (94.2%)	887 (95.2%)	0.370	561 (90.8%)	525 (86.9%)	0.032
Bad outcome (3–5)	51 (5.8%)	45 (4.8%)		57 (9.2%)	79 (13.1%)	
Alcohol intake						
Never	703 (79.5%)	708 (76.0%)	0.002	447 (72.3%)	427 (70.7%)	0.497
Current	33 (3.7%)	70 (7.5%)		20 (3.2%)	15 (2.5%)	
Past history	148 (16.7%)	154 (16.5%)		151 (24.4%)	162 (26.8%)	
Smoking intake						
Never	748 (84.6%)	753 (80.8%)	0.027	454 (73.4%)	449 (74.3%)	0.729
Current	32 (3.6%)	57 (6.1%)		16 (2.6%)	19 (3.1%)	
Past history	104 (11.8%)	122 (13.1%)		148 (23.9%)	136 (22.5%)	
Alcohol cessation						
No	847 (95.8%)	862 (92.5%)	0.003	598 (96.7%)	589 (97.5%)	0.430
Yes	37 (4.2%)	70 (7.5%)		20 (3.2%)	15 (2.5%)	
Smoking cessation						
No	849 (96.0%)	975 (93.9%)	0.036	600 (97.1%)	585 (96.8%)	0.812
Yes	35 (3.9%)	57 (6.1%)		18 (2.9%)	19 (3.1%)	
Medication non-compliance						
No	830 (93.9%)	825 (88.5%)	< 0.0001	576 (93.2%)	554 (91.7%)	0.326
Yes	54 (6.1%)	107 (11.5%)		42 (6.8%)	50 (8.3%)	
Blood pressure and fasting blood glucose						
Systolic blood pressure, mm Hg	130.82 ± 15.49	130.67 ± 13.93	0.822	130.22 ± 12.35	131.36 ± 13.87	0.133
Diastolic blood pressure, mm Hg	82.58 ± 9.48	82.08 ± 8.66	0.236	82.49 ± 7.18	83.21 ± 8.06	0.100
Fasting blood glucose, mg/dL	119.11 ± 38.88	119.51 ± 41.67	0.845	114.74 ± 38.77	115.18 ± 34.2	0.842
Lipid profile, mg/dL						
LDL	87.32 ± 32.92	86.93 ± 33.32	0.826	81.41 ± 31.19	82.36 ± 32.17	0.621
HDL	43.01 ± 13.15	42.76 ± 13.24	0.736	51.9 ± 23.97	51.72 ± 21.31	0.892
Total cholesterol	152.28 ± 37.96	153.56 ± 38.58	0.542	153.41 ± 37.71	154.36 ± 43.69	0.702
TGs	140.47 ± 70.24	142.62 ± 72.66	0.582	126.34 ± 51.90	127.57 ± 70.77	0.744
BMI and physical activity score						
BMI, kg/m^2^	25.49 ± 4.04	25.72 ± 4.90	0.275	24.05 ± 3.39	24.07 ± 3.94	0.924
Physical activity score, min	4,873.62 ± 4,081.68	4,880.13 ± 3,985.28	0.972	4,816.88 ± 3,787.52	4,404.46 ± 3,475.16	0.048

### Difference between private and government hospitals in regard to their work status prior to and post stroke

There is a significant difference between private and government hospital patients based on their work status prior to stroke (*p* < 0.0001) and post stroke (*p* < 0.0001) (Table 7). Prior to stroke, the majority of patients in both private and government hospitals were full-time paid workers [private hospitals: 871 (45.9%) vs. government hospitals: 613 (50.2%)], while the unemployed were fewer in both hospitals [private hospitals: 72 (3.9%) vs. government hospitals: 54 (4.4%)]. After the stroke, the number of unemployed people increased in both hospitals [private hospitals: 406 (22.4%) vs. government hospitals: 526 (43.0%)], while the number of full-time paid workers decreased [private hospitals: 388 (21.4%) vs. government hospitals: 84 (6.9%).

**Table 7:** Comparison of work status of patients in private vs. government hospitals before and after stroke (*n* = 3,038) status.

Categories	Before stroke		*p* value	After stroke		*p* value
	Private	Government		Private	Government	
Full-time paid work	871 (45.9%)	613 (50.2%)	< 0.0001	388 (21.4%)	84 (6.9%)	< 0.0001
Part-time paid work	70 (3.8%)	107 (8.7%)		66 (3.6%)	96 (7.9%)	
Retired	286 (15.7%)	91 (7.4%)		266 (14.6%)	72 (5.9%)	
Unemployed	72 (3.9%)	54 (4.4%)		406 (22.4%)	526 (43.0%)	
Home duties	400 (22.0%)	264 (21.6%)		241 (13.3%)	178 (14.6%)	
Student	11 (0.6%)	12 (0.9%)		10 (0.6%)	9 (0.7%)	
Others	106 (5.8%)	81 (6.6%)		439 (24.2%)	257 (21.0%)	

### Adjusted models

At 1-year, private institutions showed non-significantly lower odds of primary composite outcome [adjusted OR 0.80 (95% CI: 0.42–1.50); *p* value: 0.485] and higher odds of good functional outcomes [adjusted OR 1.91 (95% CI: 0.90–4.05); *p* value: 0.091] vs. government institutions. Private institution patients were more likely to cease alcohol [adjusted OR 3.12; (95% CI: 1.52–6.39); *p* value: 0.0002] and smoking [adjusted OR 1.99 (95% CI: 1.29–3.07); *p* value: 0.002]. There were 26% less current smoker in private institutions than government institutions [adjusted OR 0.74 (95% CI: 0.71–0.78); *p* value: < 0.0001].

In government institutions, intervention improved good functional outcomes vs. control [adjusted OR 1.56 (95% CI: 1.34–1.81); *p* value: < 0.0001]. In private institutions, intervention halved medication non-compliance [adjusted OR 0.50 (95% CI: 0.37–0.66); *p* value: < 0.0001]. There was significantly deduction in alcohol and current smoker in intervention group than control group in private institutions. The SBP and fasting blood sugar levels was decreased in intervention group in government institution patients (*p* = 0.003, *p* < 0.0001 respectively). Whereas the BMI value was decreased in private institutions in intervention group (*p* = 0.0002; Tables 8, 9).

**Table 8:** Primary and secondary outcomes (adjusted odds ratio with 95% CI).

Outcomes at 1 year	Adjusted OR (95% CI)	*P* value
Primary composite outcome		
Private hospital	0.80 (0.42–1.50)	0.485
Government hospital	Ref	
Private hospital^*^group (ref: control)	**0.87 (0.83**–**0.91)**	**< 0.0001**
Government hospital^*^group (ref: control)	1.20 (0.84–1.71)	0.304
Secondary outcome		
modified Rankin Scale (good outcome)		
Private hospital	1.91 (0.90–4.05)	0.091
Government hospital	Ref	
Private hospital^*^group (ref: control)	**0.82 (0.69**–**0.96)**	**0.017**
Government hospital^*^group (ref: control)	**1.56 (1.34**–**1.81)**	**< 0.0001**
Alcohol cessation		
Private hospital	**3.12 (1.52**–**6.39)**	**0.0002**
Government hospital	Ref	
Private hospital^*^group (ref: control)	**0.52 (0.38**–**0.72)**	**< 0.0001**
Government hospital^*^group (ref: control)	1.18 (0.79–1.76)	0.414
Smoking cessation		
Private hospital	**1.99 (1.29**–**3.07)**	**0.002**
Government hospital	Ref	
Private hospital^*^group (ref: control)	**0.60 (0.54**–**0.67)**	**< 0.0001**
Government hospital^*^group (ref: control)	0.91 (0.69–1.19)	0.481
Medication non-compliance		
Private hospital	1.57 (0.49–5.01)	0.443
Government hospital	Ref	
Private hospital^*^group (ref: control)	**0.50 (0.37**–**0.66)**	**< 0.0001**
Government hospital^*^group (ref: control)	0.79 (0.59–1.07)	0.132
Current Alcohol intake		
Private hospital	0.81 (0.57–1.15)	0.242
Government hospital	Ref	
Private hospital^*^group (ref: control)	**0.80 (0.69**–**0.92)**	**0.002**
Government hospital^*^group (ref: control)	**0.92 (0.84**–**1.00)**	**0.038**
Current smoker		
Private hospital	**0.74 (0.71**–**0.78)**	**< 0.0001**
Government hospital	Ref	
Private hospital^*^group (ref: control)	**0.74 (0.72**–**0.75)**	**< 0.0001**
Government hospital^*^group (ref: control)	1.04 (0.90–1.19)	0.614

Bold values indicate statistically significant results (*p* < 0.05).

**Table 9:** Secondary outcomes (continuous variables).

Outcomes at 1 year	β coefficient (95% CI)	*P* value
Secondary outcome		
Systolic blood pressure, mm Hg		
Private hospital	−1.097 (−2.014 to −0.180)	0.019
Government hospital	Ref	
Private hospital^*^group (ref: control)	0.192 (−2.024 to 2.408)	0.865
Government hospital^*^group (ref: control)	−1.349 (−2.260 to −0.438)	0.003
Diastolic blood pressure, mm Hg		
Private hospital	−1.041 (−1.459 to −0.623)	< 0.0001
Government hospital	Ref	
Private hospital^*^group (ref: control)	0.484 (0.344–0.624)	< 0.0001
Government hospital^*^group (ref: control)	−0.729 (−0.910 to −0.548)	< 0.0001
Fasting blood sugar, mg/dL		
Private hospital	2.185 (−0.429 to 8.670)	0.508
Government hospital	Ref	
Private hospital^*^group (ref: control)	−0.528 (−1.416 to 0.360)	0.244
Government hospital^*^group (ref: control)	−0.872 (−1.307 to −0.437)	< 0.0001
Lipid profile, mg/dL		
*Cholesterol*		
Private hospital	0.513 (−5.804 to 6.831)	0.873
Government hospital	Ref	
Private hospital^*^group (ref: control)	−1.268 (−8.500 to 5.964)	0.731
Government hospital^*^group (ref: control)	−1.020 (−6.502 to 4.460)	0.715
*Triglyceride*		
Private hospital	13.968 (3.555–24.381)	0.008
Government hospital	Ref	
Private hospital^*^group (ref: control)	−1.670 (−6.938 to 3.598)	0.534
Government hospital^*^group (ref: control)	−1.994 (−7.601 to 3.613)	0.485
*Low-density lipoproteins*		
Private hospital	5.79 (−0.457 to 16.163)	0.273
Government hospital	Ref	
Private hospital^*^group (ref: control)	0.420 (−5.321 to 6.162)	0.885
Government hospital^*^group (ref: control)	−0.707 (−5.483 to 4.069)	0.771
*High-density lipoproteins*		
Private hospital	−8.289 (−21.026 to 4.446)	0.202
Government hospital	Ref	
Private hospital^*^group (ref: control)	0.314 (−0.454 to 1.084)	0.422
Private hospital	1.165 (−0.074 to 2.405)	0.065
Government hospital	Ref	
Private hospital^*^group (ref: control)	−0.245 (−0.374 to −0.116)	0.0002
Government hospital^*^group (ref: control)	−0.020 (−0.630 to 0.590)	0.948
*Physical activity*		
Private hospital	−70.033 (−660.19 to 520.123)	0.816
Government hospital	Ref	
Private hospital^*^group (ref: control)	26.064 (−25.949 to 78.078)	0.326
Government hospital^*^group (ref: control)	340.496 (97.570–583.422)	0.006

## Discussion

This *post-hoc* analysis of the SPRINT India trial examined differences in stroke outcomes between government and private hospitals across India. Patients treated in private hospitals were more likely to be from South India and to reside in urban areas. No statistically significant difference was observed in the primary composite outcome between the two healthcare sectors at 1 year. Among secondary outcomes, treatment at private hospitals was associated with better functional recovery and higher rates of smoking and alcohol cessation.

Importantly, the comparison between hospital types was not part of the original randomization strategy and should be interpreted as exploratory. The trial was not specifically powered to detect differences between government and private institutions; thus, absence of statistical significance does not exclude potential clinically meaningful differences (Pandian et al., 2021). Despite statistical adjustment, it remains possible that baseline differences in vascular risk burden, socioeconomic status, and comorbidity profiles largely account for observed differences at 1 year.

These differences should be interpreted with caution and likely reflect underlying variation in patient sociodemographic characteristics that influence access to rehabilitation, continuity of care, and engagement in health-promoting behaviors, rather than a direct effect of hospital setting (Jones et al., 2024). Educational attainment—significantly higher among patients receiving private care and commonly used as a proxy for socioeconomic position—is associated with improved access to healthcare and better health outcomes (Jones et al., 2024; Kate et al., 2020b). Consistent with prior evidence demonstrating socioeconomic gradients in stroke outcomes in LMICs, this relationship between education, socioeconomic position, and stroke outcomes has also been reported in a dedicated SPRINT India sub-study (Lindmark et al., 2022; Marshall et al., 2015).

### Baseline risk factors, medical history, and behavioral profile

Patients managed in private hospitals exhibited a higher burden of major vascular risk factors, including HTN, DM, CAD, obesity, elevated BMI, and dyslipidaemia—features consistent with cardio metabolic syndrome. Correspondingly, these patients were more frequently prescribed antidiabetic medications and anticoagulants, reflecting both a greater documented comorbidity burden and more intensive risk stratification. In parallel, private hospital patients demonstrated higher educational attainment, urban residence, and independent home ownership, collectively indicating relatively higher socioeconomic status (SES). These findings are consistent with earlier regional analyses from the SPRINT India trial and with prior work linking education and socioeconomic factors to stroke risk profiles and outcomes (Lindmark et al., 2022; Verma et al., 2025b).

Several observed differences are influenced by the geographic distribution of participating centers. Most private hospitals (9 of 14) were located in South India, where catchment populations are predominantly urban, whereas government hospitals largely served rural communities. This distribution contributed to clear contrasts in SES, education, lifestyle behaviors, and comorbidity patterns across healthcare sectors. These institutional patterns mirror broader national trends in healthcare access and utilization. For instance, per-day hospitalization expenditure is substantially higher in private facilities (INR 6,788) than in government hospitals (INR 2,833), partially explaining preferential private-sector utilization by higher socioeconomic groups (Garg et al., 2022). Contemporary Household Consumption Expenditure Survey (HCES) data further demonstrate persistent socioeconomic gradients, with rural poverty exceeding urban poverty in 2022–2023 (7.2 vs. 4.6%; Research, 2024).

Population-level data reinforce these observations. National Family Health Survey-4 (NFHS-4) data indicate that individuals from higher socioeconomic groups account for a substantial proportion of the national burden of elevated BMI, obesity, DM, and HTN [International Institute for Population Sciences (IIPS), 2017; Corsi and Subramanian, 2019]. In parallel, a mini-review examining social determinants of obesity identifies SES—encompassing income, education, and access to resources—as a key driver, with higher burden observed among higher-SES groups in developing settings (Dinsa et al., 2012). Together, these findings suggest that the greater burden of modifiable vascular risk factors among private hospital patients reflects both socioeconomic patterning of cardio metabolic risk and potentially more systematic screening and diagnostic practices within private healthcare settings.

Stroke subtype distribution also differed between healthcare sectors. Based on OCSP classification, patients treated in private hospitals more commonly presented with partial anterior circulation syndromes and co-existing CAD, whereas those managed in government hospitals more frequently exhibited large artery atherosclerosis, TIA, and non-valvular atrial fibrillation. A higher proportion of ischemic stroke was observed among private hospital patients. Similar patterns have been reported from government medical colleges in southern India, where ischemic strokes occur at more than twice the rate of haemorrhagic strokes and anterior circulation strokes predominate—findings consistent with the South India–weighted private hospital cohort in the present study (Sridharan et al., 2009). These distributions align with the higher cardiometabolic burden observed among private hospital patients and are supported by a Gujarat-based retrospective study identifying HTN, dyslipidaemia, DM, prior CAD, and smoking as major contributors to stroke risk (Vaidya and Majmudar, 2014). National estimates of stroke burden similarly demonstrate strong associations between ischemic stroke and HTN, hyperglycaemia, and dyslipidaemia (Behera et al., 2024). Data from the Global Burden of Disease Study further indicate that individuals from higher SES, despite greater exposure to dietary and behavioral risks, experience comparatively better health outcomes and lower stroke incidence (GBD 2019 Stroke Collaborators, 2021).

Medication utilization patterns reflected these clinical and systemic differences. Patients treated in private hospitals received a higher number of medications, including antiplatelet agents, antidiabetic drugs, and anticoagulants, consistent with greater comorbidity burden and more intensive secondary prevention strategies. In contrast, patients managed in government hospitals were more frequently prescribed antihypertensive medications, aligning with higher BP levels and lower HDL cholesterol observed in this group. Comparable trends have been reported in a comparative study from Dhaka, which observed higher rates of polypharmacy in private hospitals than in government hospitals, alongside greater adherence to essential drug lists in private hospitals (Samad et al., 2024). Evidence from other LMIC settings similarly highlights constraints in drug availability and service provision in government healthcare systems, while also noting occasional overuse of medications in private care (Basu et al., 2012).

Behavioral risk profiles also differed markedly between sectors. Alcohol use, smoking, and tobacco chewing were more commonly reported among patients treated in government hospitals. National analyses identify smoking and tobacco use as leading behavioral contributors to stroke risk in India (Feigin et al., 2024). These findings are consistent with NFHS-5 and GATS-2 data, which show higher consumption of tobacco and alcohol among lower socioeconomic and rural populations—groups that predominantly access government healthcare hospitals [International Institute for Population Sciences (IIPS), 2021; Tata Institute of Social Sciences (TISS), 2018]. Additional studies confirm that both smoked and smokeless tobacco use are less common among individuals with higher educational attainment and socioeconomic position (Thakur et al., 2013). These disparities likely reflect differences in access to preventive care, health literacy, and socio-cultural practices in rural settings.

International evidence further supports these socioeconomic gradients. A 2024 Swedish stroke registry reported significantly worse outcomes among patients with lower educational attainment and income compared with those with university-level education and higher income, underscoring the consistent influence of socioeconomic position on stroke outcomes across diverse healthcare systems (Lindmark et al., 2024). Hospital sector in this analysis should be interpreted as a structural marker encompassing socioeconomic position, healthcare access, service availability, and continuity of follow-up, rather than as an independent causal determinant of outcomes. The observed differences likely reflect the complex interaction between patient characteristics and healthcare system context.

### Differential impact of the SPRINT India intervention by healthcare sector

The impact of the SPRINT India intervention varied across healthcare sectors, reflecting differences in baseline risk profiles, healthcare delivery structures, and patient engagement. Consistent with the overall SPRINT India findings—which did not demonstrate a significant reduction in the composite primary outcome but showed improvements in medication adherence and lifestyle behaviors—patients treated in private hospitals in the intervention arm exhibited comparatively better primary outcome profiles than controls. This may reflect greater responsiveness to structured secondary prevention strategies among patients with higher baseline health literacy and healthcare access. A meta-analysis of multifactorial secondary prevention programmes demonstrated greater reductions in systolic blood pressure among intervention recipients compared with controls, supporting the effectiveness of structured interventions in improving vascular risk markers following stroke or TIA (De Simoni et al., 2013).

Although patients treated in private hospitals demonstrated better functional outcomes overall, the intervention was associated with significant improvement in functional recovery among patients treated in government hospitals compared with standard care. Constraints in drug availability, rehabilitation services, and follow-up within government healthcare systems may contribute to poorer baseline functional outcomes; however, the improvements observed among government hospital patients receiving the intervention appear to be largely driven by behavioral modification. This finding aligns with systematic reviews demonstrating that adherence-focused and multimodal secondary prevention interventions improve vascular risk control and post-stroke health outcomes (De Simoni et al., 2013; Lawrence et al., 2015). These results suggest that structured secondary prevention strategies may yield meaningful functional gains, particularly in resource-constrained government healthcare settings.

Behavioral outcomes showed a clear sector-specific response to the intervention. In private hospitals, the intervention was associated with lower rates of alcohol use and current smoking, alongside a greater likelihood of cessation of both behaviors. Medication non-compliance was also reduced by nearly half among intervention recipients compared with controls. These findings suggest that patients with higher baseline health literacy and access to healthcare services may be especially receptive to adherence-enhancing interventions, highlighting the importance of sustained behavioral reinforcement even in settings with relatively robust baseline care.

Physiological responses to the intervention also varied by healthcare sector. Among patients treated in government hospitals, the intervention was associated with significant reductions in SBP and FBG, indicating improved control of major vascular risk factors. Similar benefits have been demonstrated in community-based interventions such as COBRA-BPS, which leveraged community health workers to improve blood pressure control in rural South Asia. In India, government hospitals provide free antihypertensive and antidiabetic medications through national programmes such as the National Health Mission and the National Programme for Prevention and Control of Non-Communicable Diseases, which likely synergised with the intervention to improve vascular risk-factor control (Ministry of Health and Family Welfare (India), 2023; National Health Mission (India), 2023).

In contrast, among private hospital patients, the intervention was associated with a significant reduction in BMI. Given the higher baseline BMI observed in this group, this effect may reflect better access to structured lifestyle modification resources—such as dietetic counseling and physical activity programmes—commonly available in private urban hospitals. The mHealth components of the intervention may have further amplified these advantages (Verma et al., 2025a).

Overall, these exploratory findings suggest context-specific benefits of the SPRINT India intervention across healthcare sectors—improving functional recovery and vascular risk control in government hospitals, while reinforcing behavioral change and medication adherence in private hospitals. These patterns likely reflect differences in baseline risk burden, healthcare access, and patient capacity to engage with long-term secondary prevention, rather than differences in intrinsic intervention efficacy.

### Work status, functional recovery, and socioeconomic reintegration

Substantial post-stroke employment loss was observed across both healthcare sectors. Patients treated in government hospitals experienced poorer functional recovery, contributing to a higher proportion of unemployment following stroke. While hospital type alone is not a universal predictor of post-stroke employment outcomes, the quality of care processes, access to specialized services, and patients' socioeconomic b ground play critical roles in functional recovery and workforce reintegration. Government medical institutions in India often lack structured long-term rehabilitation services, leading to persistent disability and limited functional gains. A hospital-based survey informed by the World Stroke Organization reported that dedicated stroke units, trained stroke-specific staff, and specialized diagnostic and therapeutic services were largely concentrated in private hospitals, highlighting important gaps in stroke care infrastructure within the government sector (Marshall et al., 2015).

### Strengths and limitations

Several limitations warrant consideration. A substantial proportion of participants did not complete follow-up, largely due to early termination of the trial after interim futility analysis. This attrition may have reduced statistical power and introduced potential bias. Engagement with intervention components was limited, with only 17% of participants confirming receipt of the SMS messages or videos. Such limited exposure may have reduced the measurable impact of the intervention and potentially weakened comparisons between healthcare sectors. Detailed engagement metrics stratified by hospital type were not available, limiting our ability to assess differential uptake of the digital intervention across sectors, which may have influenced observed differences (Verma et al., 2023). Although multivariable adjustments were performed, residual confounding from unmeasured sociodemographic and health-system factors cannot be ruled out. Direct measures of income, insurance coverage, discharge counseling practices, and structured follow-up systems were not systematically captured, limiting our ability to disentangle hospital-level effects from broader socioeconomic determinants. SES also intersected with educational attainment, limiting clear separation of their independent effects. Although outcome assessors were masked to treatment allocation, variation in routine clinical documentation or follow-up practices across centers cannot be entirely ruled out. Several outcomes relied on structured self-report, including medication adherence, which was not assessed using a validated adherence instrument, thereby introducing the possibility of recall and reporting bias. In addition, the predominance of urban-based centers may have limited rural representation; urban participants likely derived greater benefit from digital interventions due to higher literacy and healthcare access, while limited follow-up among rural patients—predominantly treated in government hospitals—may have led to under-ascertainment of recurrent stroke and TIA events. Despite these limitations, the prospectively defined 1-year follow-up provides valuable longitudinal data rarely available in standard care, strengthening outcome assessment.

## Conclusion

This *post-hoc* analysis of the SPRINT India trial suggests that while overall stroke outcomes are comparable between government and private hospitals, the impact of secondary prevention varies by healthcare context. Structured, low-cost interventions appear to be associated with functional recovery and vascular risk control in government hospitals and reinforce behavioral adherence in private hospitals.

## Data Availability

The original contributions presented in the study are included in the article/supplementary material, further inquiries can be directed to the corresponding author.
